# Development of High-Precision Urban Flood-Monitoring Technology for Sustainable Smart Cities

**DOI:** 10.3390/s23229167

**Published:** 2023-11-14

**Authors:** Bong-Joo Jang, Intaek Jung

**Affiliations:** Department of Future & Smart Construction Research, Korea Institute of Civil Engineering and Building Technology (KICT), 283 Goyang-daero, Daehwa-dong, Ilsanseo-gu, Goyang-si 10223, Gyeonggi-do, Republic of Korea; roachjbj@kict.re.kr

**Keywords:** disaster response, flood monitoring, IoT, pure flow energy, radar sensor, urban flood

## Abstract

Owing to rapid climate change, large-scale floods have occurred yearly in cities worldwide, causing serious damage. We propose a real-time urban flood-monitoring technology as an urban disaster prevention technology for sustainable and secure smart cities. Our method takes advantage of the characteristic that water flow is regularly detected at a certain distance with a constant Doppler velocity within the radar observation area. Therefore, a pure flow energy detection algorithm in this technology can accurately and immediately detect water flow due to flooding by effectively removing dynamic obstacles such as cars, people, and animals that cause changes in observation distance, and static obstacles that do not cause Doppler velocities. Specifically, in this method, the pure flow energy is detected by generating a two-dimensional range–Doppler relation map using 1 s periodic radar observation data and performing statistical analysis on the energy detected on the successive maps. Experiments to verify the proposed technology are conducted indoors and in real river basins. As a result of conducting experiments in a narrow indoor space that could be considered an urban underpass or underground facility, it was found that this method can detect flooding situations with centimeter-level accuracy by measuring water level and flow velocity in real time from the time of flood occurrence. And the experimental results in various river environments showed that our technology could accurately detect changes in distance and flow speed from the river surface. We also confirmed that this method could effectively eliminate moving obstacles within the observation range and detect only pure flow energy. Finally, we expect that our method will be able to build a high-density urban flood-monitoring network and a high-precision digital flood twin.

## 1. Introduction

Owing to global warming, climate change, including rising ocean temperatures and outbreaks of extremely severe weather, is increasingly occurring worldwide. Large-scale storms such as typhoons and hurricanes occur frequently, whereas incidents of sudden heavy and local torrential rainfall, which were previously uncommon, are increasing exponentially. Consequently, record-breaking rainfalls have been observed in major cities with high population densities worldwide, resulting in severe casualties. In particular, during the summer of 2020 in South Korea, the longest rainy season in history (54.3 d) was recorded, and the national average precipitation during the rainy season was 687 mm, which is nearly twice the average during the same period in different years [1]. Numerous disasters have occurred; in particular, flood damage during the summer worsened as Typhoons Maysak and Haishen hit the Korean Peninsula. At least 50 people were killed or missing [2], and severe damage occurred as rivers flooded and banks were lost in major basins throughout Korea, including the tributaries of the Hantangang, Seomjingang, Hapcheon Hwanggang, and Nakdonggang rivers [3,4]. Furthermore, damage of various magnitudes has been reported across the country. To prevent flood disasters due to climate change, many countries or cities have introduced early warning systems; however, the scale and frequency of damage from flood disasters are sharply increasing globally.

Rapidly developing cities often face difficulties in expanding their drainage facilities despite the frequent heavy rainfall every year, thus experiencing severe damage from inundation. In 2020, three people died in an underpass in Choryang-dong, Busan, because of flooding [5]. In August 2022, a family of three living in a semi-underground housing unit in Sillim-dong, Seoul, was killed owing to flooding caused by heavy rainfall; further, nine people died in an underground parking lot in Indeok-dong, Pohang, which was suddenly flooded by rainfall as Typhoon Hinnamnor hit Korea in September 2022 [6]. These incidents prove that flood damage induced by heavy rainfall can occur in rural areas, river basins, and urban areas.

**Figure 1:**
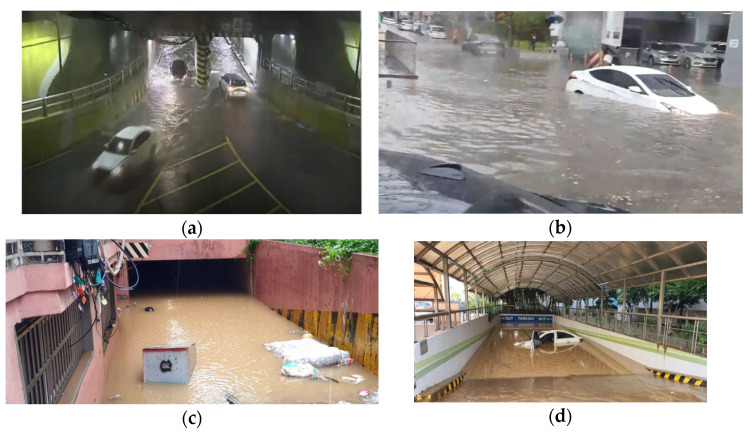
Cases of flooding in lowlands: (**a**) underground road in Busan [7]; (**b**) underground road in Cheonan [8]; (**c**) underground house in Sillim-dong [9]; (**d**) underground parking lot of apartment in Pohang city [10].

In particular, the risk of flooding in cities is high because of the fast water flow on well-paved roads or in residential and living areas can cause people to fall or drift away easily, even at a low water level, owing to the lack of obstacles that can reduce the water velocity. Furthermore, rainwater overflowing from drainpipes does not permeate the ground, and instead, flows into the lowlands, thus causing sudden flooding. The probability of people being isolated owing to the characteristics of buildings or complex urban structures is high, even when the flood level is low. Figure 1 lists actual flood sites that have recently occurred in Korea as examples of flash flood risk in urban areas.

Accordingly, the national and local governments of Korea are striving to prevent flooding by properly maintaining drainage facilities during summer to mitigate casualties from urban flooding. An urban flood-monitoring system operates for urban rivers based on various data types, such as flood monitoring, rainfall observation, rainfall forecasting, and closed-circuit television (CCTV) footage [11]. However, monitoring the flooding status of lowland houses, underpasses, underground parking lots, and road culverts at the individual level is practically impossible. Furthermore, determining the appropriate time for evacuation or risk notification is even more challenging considering the nature of urban flooding, where the water level rises extremely quickly.

Even in cities designed to accurately predict such risks based on long-term statistics and population changes, ensuring safety from unpredicted natural disasters such as sudden downpours remains an issue. Old cities that have developed over a long period typically have various underground facilities, such as electricity, gas, water supply and drainage, and hot water pipes, entangled in a complicated network; thus, actualizing a city safe from flooding is challenging unless the city is redesigned from the start.

The United Nations Economic Commission for Europe (UNECE) defines a sustainable smart city as an innovative city that fulfills the demands of current and future generations in terms of economy, society, environment, and culture while improving quality of life, city operation, service efficiency, and competitiveness by utilizing information and communication technology and other relevant measures [12]. In a UNECE report [13], hazard management was designated as the basic element of a city necessary to comprehensively foster sustainability and a livable life. Hazard management in a sustainable city requires preventive solutions for natural, technological, and comprehensive risks and preparation and emergency response actions. Most cities are particularly vulnerable to disasters because people, housing, and capital are concentrated in urban areas. Cooperation among various institutions is required to create resilient cities in these environments. In other words, cooperation must be exercised to prevent disasters by strengthening the internal competence of urban systems, minimizing negative impacts in the event of a disaster, and mediating restoration activities after a disaster.

Recently, Ramos and Besharat [14] compared different scenarios related to flood risks and the efficiency of sustainable drainage systems and proposed a scenario that minimized flood risk and the effects of extensive urbanization from the perspectives of economic feasibility and hazards. Li et al. [15] analyzed the urban flooding mechanism by connecting climate change with urban river networks and urban facilities. They proposed various methods for evaluating flood risk. Their proposed scenarios combined two measures: the retention basin and permeable road pavement of a sustainable city. However, these methods require long-term urban planning and growth, which is impractical for immediate responses to extreme weather conditions.

Taromideh et al. [16] proposed a method in which administrators and decision-makers can accurately determine flood risk by integrating machine learning (ML) and a semi-subjective analytic hierarchical process in regions where the collection of statistical data is challenging, such as developing countries. However, this method is based on macroscopic flood risk assessment criteria suitable for a city-level scale, and thus, is challenging to apply to the actual risks occurring in complex urban areas.

To implement a safe smart city with sustainability and resilience to frequent weather damage, this study proposes a technology to directly monitor flooding status and issue notifications in real time. The proposed system was installed in lowland houses and underground facilities (underpasses, underground parking lots, and road culverts) vulnerable to flooding. The proposed technology can precisely distinguish the inflow of rainwater from the surrounding environment, which changes rapidly, by utilizing industrial radar and signal-processing algorithms. It calculates the inflow velocity of rainwater and flood level in units of 1 cm. Unlike previous indirect flood analysis methods that depend on CCTV footage or the amount of rainfall, the proposed technology can respond immediately by directly observing the water level at the site. Therefore, our proposed method is expected to contribute significantly to the urban flooding response, as it can promptly and accurately provide flooding information in emergencies.

The remainder of this paper is organized as follows. Section 2 reviews existing technologies related to the technology proposed here. Section 3 provides a detailed description of the proposed method. Section 4 describes the experimental setting designed to validate the proposed method and flood-monitoring results. Finally, Section 5 summarizes the findings and concludes the paper.

## 2. Literature Review

### 2.1. Urban Flood Management

The Han River Flood Control Office, under the supervision of the Ministry of Environment [11], intensively monitors national streams and regions with high population densities. The Han River Flood Control Office established a flood-season response system to forecast floods, manage quality, and propagate forecast information before the occurrence of damage. The Han River Flood Control Office monitors major rivers and streams prone to flooding in real time, thus establishing a more effective proactive response system.

Recently, several studies have investigated methods for determining flood risk by employing ML [17,18,19,20]. Park and Kim [17] built an artificial intelligence (AI) model that utilized recurrent neural networks to predict river flooding by measuring the water level at two upstream points. They applied an AI model to predict the water level of rivers; however, the dependence on the performance of a water level measurement instrument is extremely high, and its applicability to real-time warning notification systems for sudden flooding is yet to be validated.

Another study utilized a hydraulic model to calibrate and validate previous flood events to estimate the appropriate roughness coefficient for various flood discharge conditions. A method was established to extrapolate a water level–flow velocity curve by adopting a calibrated and validated model [21]. This method estimates the unmeasured sections by utilizing extrapolations when the flow velocity measurements have missing values. Therefore, this method is limited in terms of the immediate response to sudden floods caused by local torrential rainfall.

As mentioned earlier, national rivers and major cities with water basins or streams have well-established flood forecasting or warning systems, which allow for quick, responsive actions to secure the optimal time for evacuation in urgent situations and prompt recovery through national resources. However, in the case of inundating lowlands or road culverts in cities, the technology applied to rivers cannot be readily utilized in complex urban environments.

Guo et al. [21] investigated various river flooding models for predicting urban flooding. When a river flooding dynamic model is applied to urban flooding and inundation, several numerical issues arise due to the representation of urban terrain, processing method of urban river water, calculation efficiency, and complicated environmental characteristics. Xia et al. [22] introduced a numerical system to model the flow of river flooding on complicated ground terrain to maintain numerical stability and prediction accuracy. Unlike common river basins, urban regions comprise complicated terrain and underground infrastructures, significantly influencing urban flooding. To overcome this problem, a porosity-based shallow water equation (SWE) model is proposed to generalize the effects of high-density buildings within a city. This model focuses on numerical analysis to solve the urban flooding dynamics caused by the water inflow of rivers around cities rather than on the direct influence of heavy rains. Hence, the practical application of this model to urban flooding caused by heavy downpours is challenging.

Glenis et al. [23] proposed a drainage simulation module and a two-dimensional SWE model for major buildings and city infrastructure based on a high-resolution digital elevation model (DEM). Owing to the increased calculation complexity of street-level or meter-scale high-resolution models, various technologies, such as graphics processing units, distributed processing, and parallel algorithms, are required to improve speed.

Despite such attempts, considering all the details of urban environments that may influence flooding caused by heavy rain or overflows remains challenging because urban environments experience rapid transformation. Accordingly, their reliability and efficiency require improvement considering the development of currently available urban flooding models. As mentioned earlier, urban environments have complex underlying surface characteristics, and abrupt changes occur when applying the model because of the nature of cities. Therefore, most numerical models often employ empirical equations that lack stability and accuracy or directly utilize simplified methods.

To reduce the risk of sudden urban flooding caused by heavy downpours, a novel urban flooding prediction model must be developed based on direct and immediate flood-monitoring technology with high accuracy, and the data obtained from such technology should be utilized rather than relying on previous model-based flood-risk-judgment and -prediction technologies.

### 2.2. Urban Flood-Monitoring Technology

As previously explained, general flooding and overflow modeling was performed based on a model that utilizes the flow of water and its geographical characteristics. More detailed geographical data and drainage network information are utilized in urban regions.

This section presents different measures for utilizing flood models or directly monitoring risks by measuring or observing the occurrence of flooding. Because urban flooding mainly results from heavy rain, the amount of rainfall is utilized as an input parameter when developing flood prediction models. The amount of rainfall was computed using a rain gauge, weather radar, and satellite images and was applied as the representative rainfall value in each domain of the geographical model.

Elkhrachy [24] applied an ML regression algorithm that utilized several remote sensing datasets, including those from the Sentinel-1 and Sentinel-2 satellites, and a digital surface model to detect unexpected flooding. The flood water depth was estimated based on ML by detecting the edges and key point features utilizing remote sensing SAR images. When various ML techniques were applied in Cairo, Egypt, the RMSE accuracy ranged from 0.18 to 0.22 m at a depth of less than 1 m. However, the utilization of macroscopic methods based on satellite images and ML results in less accurate observations and predictions in urban environments, which are complicated and change frequently. This technique is also based on a flood model explained in the previous section; thus, it cannot be practically applied as a direct monitoring and warning method in flood-prone areas in cities.

To reduce flood damage that occurs suddenly in cities with high population density and floating populations, the most accurate method is to directly monitor flooding for sites with a high possibility of flooding. Urban flood-monitoring equipment can be categorized into contact and non-contact types depending on whether the equipment is in contact with water. The contact-type equipment, which is most commonly utilized, includes float, reed, pressure, and bubble types. Recently, non-contact equipment has been utilized, including sound waves, ultrasonic waves, radar, LED elements, and CCTV footage. Table 1 presents detailed descriptions of the various types of water meters [25].

Conventional non-contact-type water level gauges/velocimeters are mostly developed for installation in rivers or dams with high flow volumes; thus, they are difficult to install in urban ecological streams, roads, and lowlands that are exposed to interference caused by changes in the surrounding environment. Conventional contact-type water level gauges/velocimeters result in poor management efficiency if installed and operated in places that do not often flood or require additional infrastructure, and cannot be relocated easily if installed at urban drainage facilities.

Surface image velocimeters (SIVs) involve the installation of a surveillance camera at rivers, and the computation and calibration/validation of the moving speed of a tracking particle by setting a reference point in the images and letting the tracking particle flow in the absence of rainfall [26]. SIVs are sensitive to changes in the environment of rivers, such as illuminance and shade, and entail increased observation errors based on the assumption that the river surface undergoes relatively few changes. Furthermore, this equipment cannot be utilized in dry urban areas.

Owing to the significant evolution in computational speed and deep learning technology since 2010, studies on urban flooding utilizing non-contact methods, such as CCTV footage, have been conducted [27,28,29,30,31]. Qiao et al. [28] proposed a deep learning-based (YOLOv5s and convolutional neural network (CNN)) method to measure the water level to resolve the problem of poor scene adaptability and weak robustness, which is a drawback of conventional water level gauge reading methods based on images. However, this method is ineffective in places without rivers because it involves reading the number of water level indicators in a river.

Lopez-Fuentes et al. [29] proposed a multimodal deep learning model to detect flooding in social media posts; this model comprised a CNN, which extracted visual features and a bi-directional long short-term memory network, which extracted semantic features from text metadata. Han et al. [30] proposed a puddle detection method based on reflection attention units and a fully convolutional network. Sarp et al. [31] proposed an automatic overflow detection and segmentation method utilizing a Mask-R-CNN, a type of region-based CNN, for object detection and semantic segmentation. Most deep learning algorithms that employ CCTV footage are aimed at crime prevention and security; thus, they cannot be effectively utilized to detect rainwater flowing into lowlands. Other issues, such as glare, flare, and ghosting in cameras, may result in poor resolution. Heavy rain and trees may block the view, or image quality can be significantly degraded at night, which will also cause difficulty in determining flooding.

Thus, this study proposes a novel technology that can build a more efficient observation network than conventional flood-monitoring technologies and directly determine flood risk compared with previously explained prediction and estimation methods. The proposed technology is designed to discern only pure flow energy by effectively filtering factors hindering flood-monitoring accuracy, such as humans, cars, animals, and plants, in urban ecological streams, lowlands, underpasses, and underground facilities with complicated environments and surrounding noise. It was developed as a miniaturized Internet of Things (IoT) sensor platform to facilitate installation and operation.

**Figure 2:**
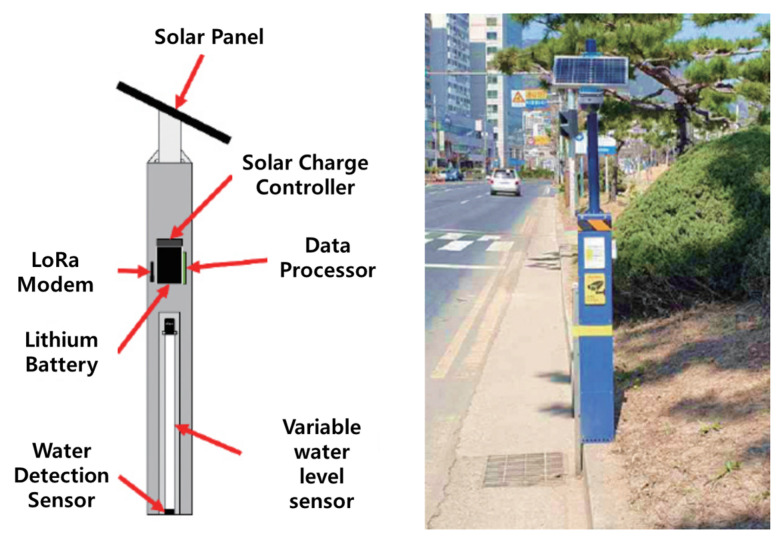
Concept and installation example of urban flooding sensor currently in operation in Republic of Korea [32].

To explain the differentiation of the proposed technology in more detail, the urban flooding sensor currently in operation in the Republic of Korea [32] is shown as a conceptual diagram and an example of an actual installation site in Figure 2.

The urban flooding sensor shown in Figure 2 is a technology developed and currently in operation by the National Disaster and Safety Research Institute. Much research is being done to use the data observed from these sensors to determine the presence or absence of flooding in a specific location and use it as the basis for an urban flood model. However, as can be seen from the field-installed sensor device in Figure 2, this method obtains a response by directly exposing the sensor to water, so the observation range and installation range for flooding are extremely limited. In addition, because the structure of the sensor requires direct contact with water, there is a high possibility of errors due to trash or floating objects, so continuous maintenance is essential. Additionally, because it must be installed in the form of a pole, there are administrative and legal restrictions on the installation of public infrastructure. Crucially, it is difficult to install near urban rivers where inland water flooding is likely to occur. So, when a river floods and sensors detect flooding, the risk level may already be high. In addition, because it cannot detect the flow rate or speed of flood water, it is difficult to expect that it will be of much use in responding to and predicting water disasters.

Therefore, in this paper, we propose a new type of urban flood-monitoring technology to overcome as much as possible the problems with the existing technologies listed above.

## 3. Proposed Urban Flood-Monitoring Technology

### 3.1. Overview of Technological Development

**Figure 3:**
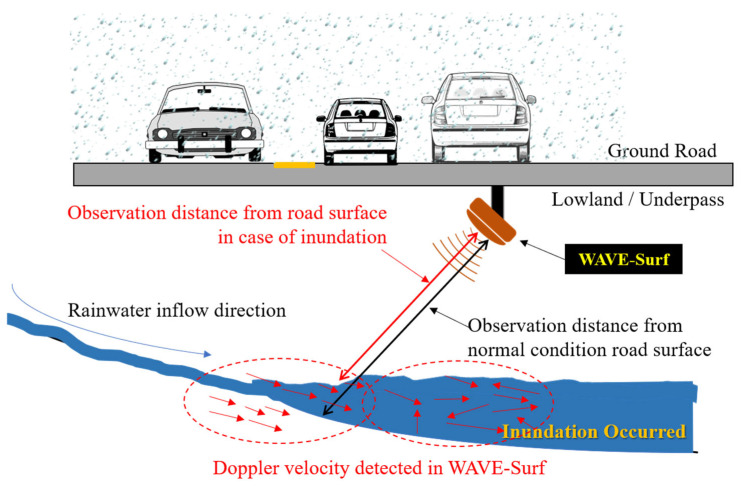
Concept and simple example of proposed WAVE-Surf system.

The proposed water level and velocity surveillance system for urban and road flooding (WAVE-Surf) is a subminiature low-power IoT technology based on an industrial radar sensor that can be easily installed in lowlands or underground facilities in cities. A schematic of the proposed technology and a simple example are presented in Figure 3.

Figure 3 illustrates a situation in which flooding occurs as rainwater flows into an underground facility or underpass. In this example, WAVE-Surf is installed at a point of rainwater flow or at the lowest point where flooding may occur, thus monitoring rainwater inflow and flooding in real time. WAVE-Surf detects flooding utilizing the signals of an industrial radar sensor, thereby calculating the flood level and inflow velocity by analyzing the radar reflectivity signal. When WAVE-Surf is operated, as illustrated in the figure, the Doppler velocity of the radar signals for rainwater flow is generated when the rainwater flows in, and the amount of rainwater inflow can be determined based on the intensity and distribution of the Doppler velocity. When flooding occurs, the radar reflectivity distance decreases owing to the flood level, and surface flow velocity is generated in the flooded region. Therefore, the risk was determined by calculating the flooding depth. The detailed algorithm of the flood-monitoring technology is explained in Section 3.2.

### 3.2. Development of Urban Flood-Monitoring Technology

The WAVE-Surf proposed here was designed as a low-power IoT sensor platform based on a subminiature industrial radar with a bandwidth of 77 GHz. Urban rivers, lowlands, underpasses, and underground facilities have high human and vehicle traffic volumes, and various devices and machines are constantly in operation. Thus, WAVE-Surf was applied to different algorithms utilizing radar observation values to accurately judge the flood risk and detect the inflow of rainwater and flood levels.

**Figure 4:**
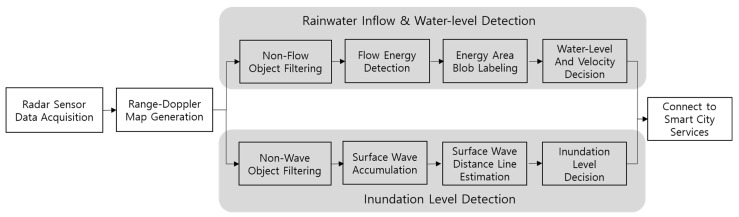
Functional block diagram of WAVE-Surf system.

A schematic of the algorithm executed by WAVE-Surf for real-time flood monitoring is presented in Figure 4, in which WAVE-Surf is divided into two parts according to the flooding type. The first technique involves the detection of rainwater inflow velocity, flood level, and flooding velocity in real time by directly monitoring the rainwater that flows abruptly into the lowlands. The second technique involves detecting the flood level, provided the rainwater inflow cannot be directly monitored.

**Figure 5:**
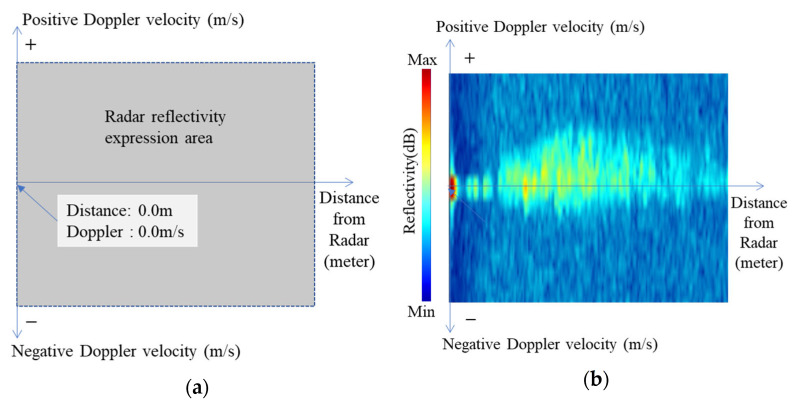
Range–Doppler map of FMCW radar data: (**a**) meaning of each axis and field on map, (**b**) example of map representation of radar data in one frame.

To detect rainwater inflow and flood levels in cities, WAVE-Surf is equipped with a frequency-modulated continuous wave (FMCW) radar sensor that utilizes a bandwidth of 77 GHz and is commonly utilized for short-distance movement detection, altimeters, and autonomous driving. First, the proposed method obtains the reflectivity and Doppler velocity with respect to the distance from the FMCW radar and subsequently generates a two-dimensional graph image through range–Doppler mapping. The utilization of graph images allows the connectivity between continuous or discrete Doppler velocities to be intuitively understood and the flow energy of rainwater to be easily separated from the surrounding objects. A representation and an example of a range–Doppler map are presented in Figure 5.

The range–Doppler map, represented in terms of the Doppler velocity distribution (y-axis) with respect to the radar reflectivity distance (x-axis), is presented in Figure 5a, where the reflectivity of an object detected by the radar in the radar reflectivity expression area is expressed on the map along with the Doppler velocity of the object. The range–Doppler map of an actual river surface observed during one frame from the radar is displayed in Figure 5b, where the blue color represents low reflectivity, and the red color represents high reflectivity. The reflectivity in this graph has a positive Doppler velocity in most distant areas because the flow (by the positive Doppler velocity) toward the radar was detected over a long distance at a very close distance from the river surface. Rainwater inflow and flood level detection were performed using a range–Doppler map.

#### 3.2.1. Rainwater Inflow Detection by Utilizing Flow Energy Detection

As mentioned in the previous section, complicated urban environments, including lowlands and underground facilities, involve frequent movements of humans, bicycles, and motor vehicles. Such objects are frequently detected by a radar sensor when utilized to monitor rainwater inflow; as these objects have strong reflectivity and high Doppler velocity, they can significantly hinder the accuracy of detecting the inflow of rainwater, which has relatively weaker reflectivity and Doppler velocity, or cause misdetection.

To overcome this issue, a method for detecting rainwater by extracting the flow energy of the rainwater inflow through signal processing was applied here.

From the radar perspective, the monitoring distance of a moving object generally varies depending on its mobility; the Doppler velocity changes or even becomes the opposite, thus demonstrating high variation characteristics within a range–Doppler map.

In contrast, flooding is not caused by momentary rain but by a continuous inflow of rainwater, even for a short period; thus, the continuous Doppler velocity and direction of rainwater flowing into the lowlands are observed at the same point (or distance) within the range–Doppler map. Jang et al. [33] proposed a method for monitoring the overflow of small streams and valleys in rural areas, which was applied here to detect the rainwater flow.

**Figure 6:**
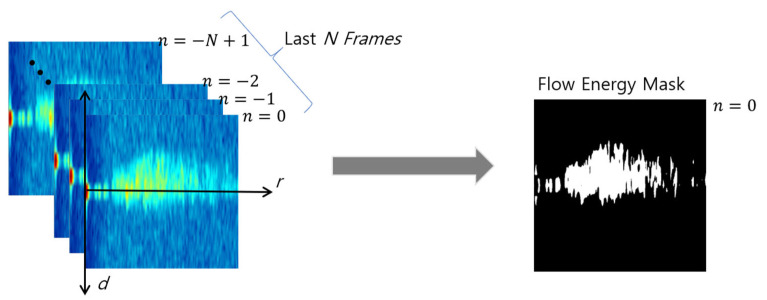
Flow energy mask for water-flow detection.

As proposed by Jang et al. [33], WAVE-Surf determines the presence of flow energy at a coordinate (r, d), provided all reflectivity values $z_{n}(r,d{)}_{\;}$, (−N<n≤0) at the same coordinate (r, d) on a range–Doppler map satisfy the reflectivity threshold ${th}_{Z}$ for the latest N number of frames detected by the radar, to detect pure water flow in a complicated environment. Similarly, the flow energy mask M can be generated in the entire range of the Doppler map, and the mask value for each coordinate is defined in Equation (1).

(1) $$m_{n=0\;}\left(r,d\right)\left\{\begin{array}{cc} =\;True, & \;\;if\;\left(z_{n}\left(r,d\right)>{th}_{Z}\right)\;for\;\left(-N<n\leq 0\right)\\ =\;False, & \;\;otherwise \end{array}\right.,$$

where n=0 represents currently monitored frames and masks. The process of generating a flow energy mask utilizing Equation (1) to extract pure flow energy is illustrated in Figure 6.

As previously mentioned, this study applied a statistic filtering technique in which the probability of reflectivity and Doppler velocity of non-water-flow objects that are continuously observed at one point is low; in contrast, water-flow energy continuously has a similar Doppler velocity at the same point, while N number of data are observed. Here, the number of frames required to generate a mask, N, and the reflectivity threshold, ${th}_{Z}$, of the water flow can be empirically set according to the condition of each site, considering the environmental characteristics (underpass, stairs, and underground parking lot) and the distance from the ground.

**Figure 7:**
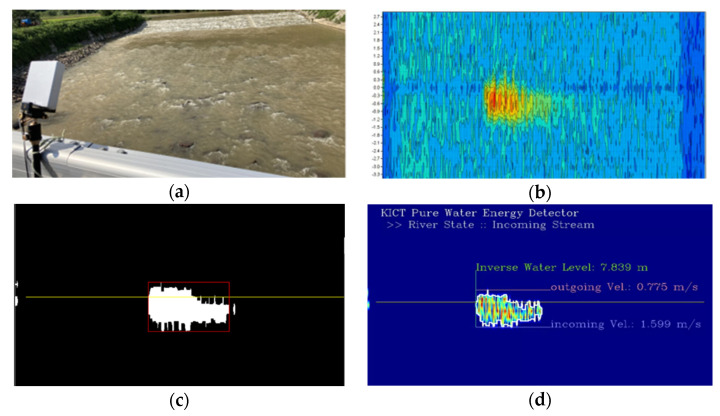
(**a**) On-site water monitoring utilizing WAVE-Surf; (**b**) actual range–Doppler map observed from (**a**); (**c**) blob-labeling result (red box) and flow energy mask generated from (**b**) (N = 3); and (**d**) calculated water level and velocity from (**c**).

For the ultimate detection of water-flow energy, a blob-labeling algorithm [34] was applied to cluster the connected coordinates, detect the distance of water flow from the cluster closest to the radar point, and determine the water-flow velocity from the cluster with the largest (negative or positive) Doppler velocity. The results of blob labeling from the currently observed frame upon utilizing M are presented in Figure 7. The number of consecutive frames is set to N = 3.

**Figure 8:**
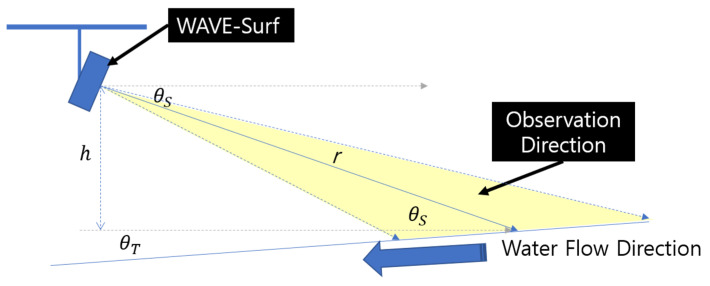
Environmental variables for water-level and velocity correction in environment where WAVE-Surf is installed.

Figure 7a illustrates that the water level and velocity must be corrected based on the WAVE-Surf installation angle and ground slope. The default water level and velocity can be identified based on Figure 7, from which the corrected water level and velocity can be calculated. A schematic of the relationship between distance r from angle $\theta_{S}$ in which WAVE-Surf is installed horizontally, vertical distance $h_{}$ from the observation point, and ground slope $\theta_{T}$ is presented in Figure 8.

First, the vertical distance r from where flow energy is detected to the WAVE-Surf observation point, h, can be easily calculated based on the WAVE-Surf observation angle, $\theta_{S}$, by utilizing Equation (2).

(2) $$h=r•sin\theta_{S}$$

Suppose that the vertical distance from the ground or normal water level to the WAVE-Surf observation distance $r_{0}$ is $h_{0}$ when flooding is not considered. In that case, $h_{I}$ or

(3) $$h=h_{I}-h_{0}=(r-r_{0})∙sin\theta_{S}$$

can be applied to calculate the flood level or elevated water level.

Unless a valley or waterfall is being analyzed, the slope of the urban river surface over a short distance is $\theta_{T}\approx 0$ and can be neglected. However, for urban lowlands or slopes that are rarely flooded, the horizontal velocity $\theta_{T}$ of the ground must be considered to correct the rainwater inflow velocity. The corrected velocity $v^{\prime }$ can be calculated by employing Equation (4) from the velocity v presented in Figure 7:

(4) $$v^{\prime }=v•sin(\theta_{S}+\theta_{T}),\;\;\;\;\theta_{S}+\theta_{T}\neq 90$$

The flood risk can be estimated by utilizing the approximate distance and velocity data obtained from the flow information, as presented in Figure 7. However, the flood level and velocity were corrected using Equations (2)–(4) to provide the optimal flood risk information of a site by accurately calculating the flood level and rainwater inflow velocity at an observation point. Finally, the flood level $h_{I}$ and rainwater inflow velocity (or surface velocity) $v^{\prime }$ were automatically calculated immediately after the detection of water energy by WAVE-Surf and sent to a control center or utilized to calculate the risk level for direct notification. Based on these results, the rainwater and flood levels and the velocity at which they approach or recede can be determined.

#### 3.2.2. Flood-Level Detection by Utilizing Scattering Waves

**Figure 9:**
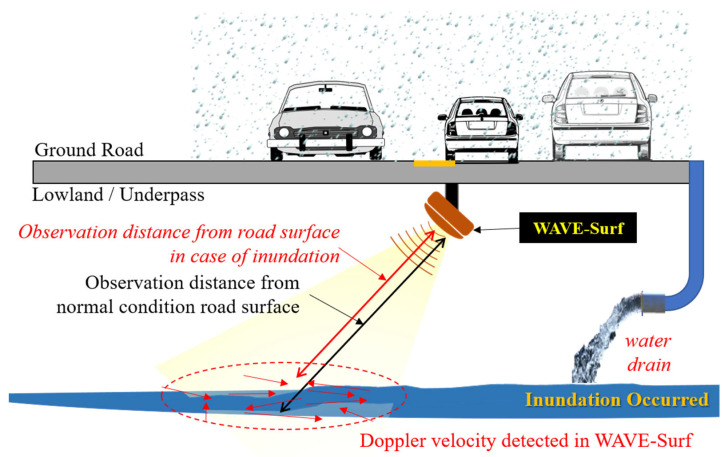
Example of situation in which flooding occurred at WAVE-Surf non-monitoring point.

As described in the previous section, we examined a method for detecting rainwater inflow utilizing flow energy detection. The previous method judged the risk because WAVE-Surf detects the signal from the flow energy of the inflow of rainwater. However, the method explained in the previous section can only be applied to sections in which continuous flow occurs. In other words, detecting the flood level may be challenging if WAVE-Surf monitors a region without the flow of water (or rainwater inflow point), as illustrated in Figure 9.

If flooding occurs at a point not monitored by WAVE-Surf, as illustrated in Figure 9, the flood level can be challenging to detect by utilizing the flow energy detection method explained in the previous section, provided that flooding occurs in the absence of evident flow energy in one direction. This section proposes a method for effectively detecting flood levels in real time in such cases.

Furthermore, this method utilizes the range–Doppler map observed in WAVE-Surf, as illustrated in Figure 5. The two algorithms were executed simultaneously for a range–Doppler map of one frame.

All values on the range–Doppler map in Figure 5 theoretically converged to zero on a road surface without flooding. When flooding occurred, as illustrated in Figure 9, the inflow of water generated irregular wavelengths at the water surface in a stagnant state; thus, WAVE-Surf could detect objects, such as noise, with a Doppler velocity in all observation areas of a range–Doppler map in both positive and negative directions in a low Doppler velocity range.

**Figure 10:**
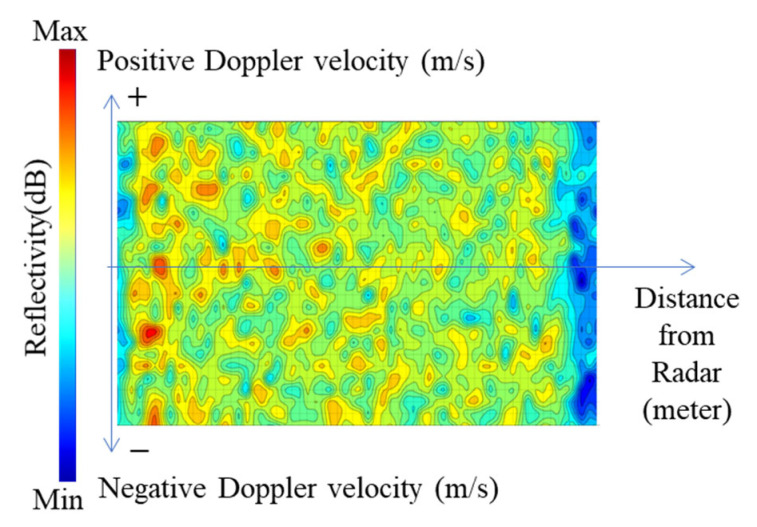
Observation characteristics of WAVE-Surf range–Doppler map based on irregular waves generated from water surface.

The results of observing the water surface wavelengths from a close distance using WAVE-Surf are illustrated in Figure 10. A weak Doppler velocity was detected for the scattered waves throughout all areas, excluding certain areas at the closest and farthest distances on the range–Doppler map.

A mask can be applied to the scattering waves for flood detection without flow, as demonstrated in Figure 10.

First, most scattering waves observed during flooding have a gentle waveform and a lower Doppler velocity than nearby objects. Therefore, coordinate values $z^{\prime }(r,d)$ with a Doppler velocity lower than the threshold were extracted by utilizing the maximum threshold of the Doppler velocity for all reflectivity values present at the coordinate (r, d) in Equation (5) on the range–Doppler map in Figure 10.

(5) $$z^{\prime }(r,d)=\left\{\begin{array}{cc} z(r,d) & ,\;if\;|z(r,d)|\;<\;{th}_{D}\;\\ 0\;\;\;\;\;\;\;\; & ,\;otherwise \end{array}\right.$$

For $z^{\prime }(r,d)$ filtered for Doppler velocity, the minimum and maximum thresholds, ${th}_{W1}$ and ${th}_{W2}$, of reflectivity, respectively, which were empirically determined, were applied to create a scatter mask, b(r,d). The minimum threshold ${th}_{W1}$ of the reflectivity was utilized to minimize the impact of the radar noise signal. As the reflectivity generated by waves during flooding is lower than that of other nearby objects, non-water objects can be filtered by filtering reflectivity exceeding a specific threshold, ${th}_{W2}$.

(6) $$b(r,d)=\left\{\begin{array}{cc} 1 & ,\;\;\;if\;\;\;\;\;{th}_{W1}<z^{\prime }(r,d)<{th}_{W2}\\ 0 & ,\;\;\;otherwise \end{array}\right.$$

To calculate the flood level utilizing the scatter mask b(r,d) extracted from the range–Doppler map, the latest N observation frames were utilized to perform a logical OR operation at the same coordinates to create a flood-level decision map D(r,d), as expressed in Equation (7).

(7) $$D(r,d)=\;b_{n}(r,d)\;\;OR\;\;\;b_{n+1}(r,d)\;\;OR\;\;\;\;\ldots \;\;\;OR\;\;\;\;b_{0}(r,d)\;$$

**Figure 11:**
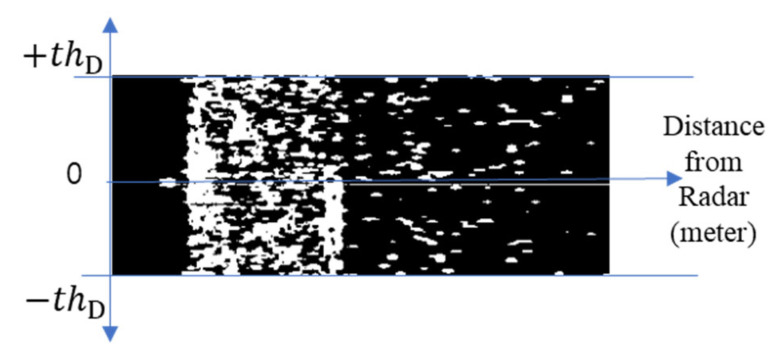
Flood-level decision map generated from successive WAVE-Surf range–Doppler maps.

An example of a flood-level decision map D(r,d) observed in WAVE-Surf is presented in Figure 11. Here, N = 5 and ${th}_{D}=0.2\;m/s$.

**Figure 12:**
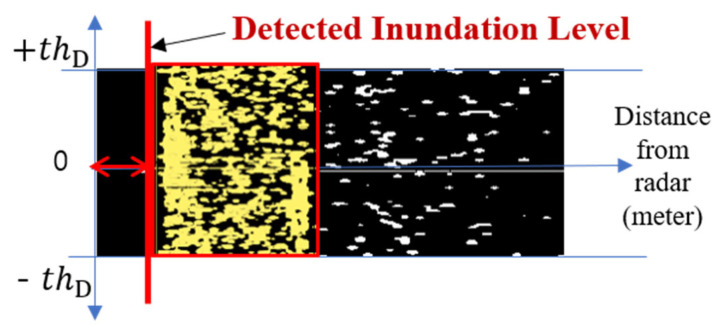
Flood decision process utilizing flood decision map.

Finally, a flooding distance boundary can be obtained by performing blob labeling for bright regions, as in the previously explained flow energy detection, from the flood decision map, D(r,d), as valid wave information. The flood information determined by the bounding box of the blob-labeled area in Figure 1 is presented in Figure 12.

## 4. Experimental Results

To verify our proposed technology, various experiments were performed indoors and in real river environments. In the indoor experiments, time delay and detection accuracy were measured by detecting water level and flow velocity in a narrow and complex place with many obstacles. Since it was difficult to artificially control the water level and flow rate in experiments in outdoor environments, the accuracy was verified by measuring the distance and flow rate between the developed WAVE-Surf and the water surface by diversifying the observation locations.

### 4.1. Experimental Results in Indoor Environments

In an indoor experiment, a device that artificially generated a flow of water was manufactured to simulate the flooding of low-lying facilities or underpasses in the city. Using this device, experiments were performed in the laboratory environment with many narrow and complex structures.

#### 4.1.1. Indoor Experimental Equipment Configuration

**Figure 13:**
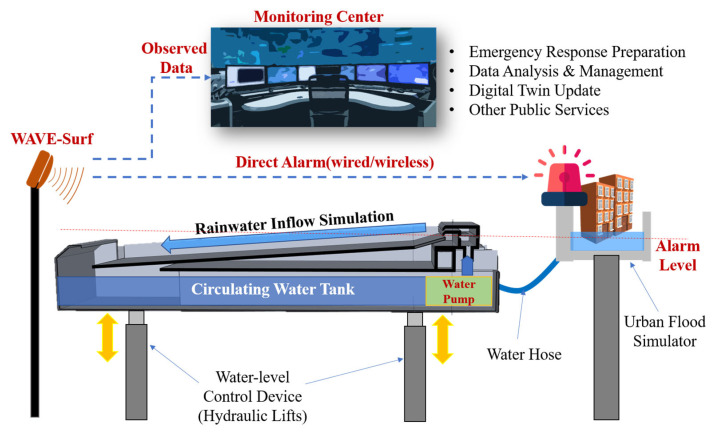
Experimental environment for urban flooding simulation.

An indoor simulation environment was configured, as illustrated in Figure 13, to validate the sustainable and secure smart city’s flood prevention system, which was this study’s original objective.

The experimental environment in Figure 13 was set to facilitate experimentation in various situations, because conducting an experiment in actual flooding situations can be problematic in terms of safety and scheduling. A water tank was fabricated to create water flow by utilizing a water pump to simulate rainwater inflow and drainage. Hydraulic lifts that could adjust the height in 1 cm units were installed underneath the circulating water tank to remotely move the tank up and down to simulate the rise and fall of the flood level.

**Figure 14:**
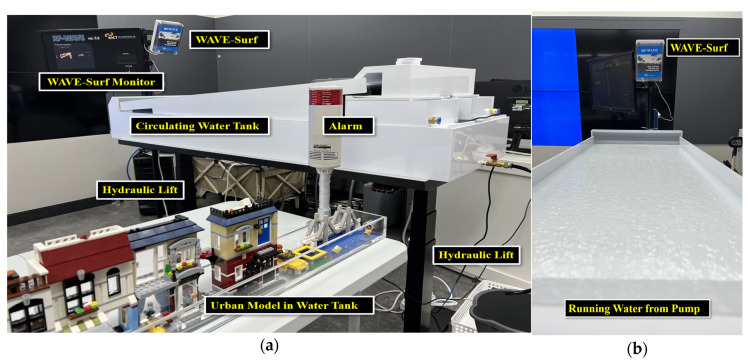
(**a**) Implementation of urban monitoring environment for WAVE-Surf experiments, and (**b**) simulating a flood.

Furthermore, a city model was fabricated in a separate water tank at an appropriate height and connected to the circulating water tank through a hose; thus, flooding was simulated as water flowed into the city model when the circulating water tank rose above a certain height, $h_{Alarm}$. WAVE-Surf monitored the circulating water tank, which was considered an urban lowland, and immediately operated an emergency alarm device when the height of the circulating water tank exceeded $h_{Alarm}$ (urban flood risk height) according to the previous setting. The actual experimental environment, configured based on the conceptual diagram in Figure 13, is presented in Figure 14. The actual water flow is presented in Figure 14b.

#### 4.1.2. Indoor Experimental Results

In general, underpasses, low-lying facilities, and building basements are kept dry. If flooding occurs due to heavy rainfall or water-related incidents, flow velocities will occur at the points where water enters these low-lying areas. Experiments were conducted using the simulation device in the previous section to determine whether the proposed technology could be used to accurately observe the start of the flood.

**Figure 15:**
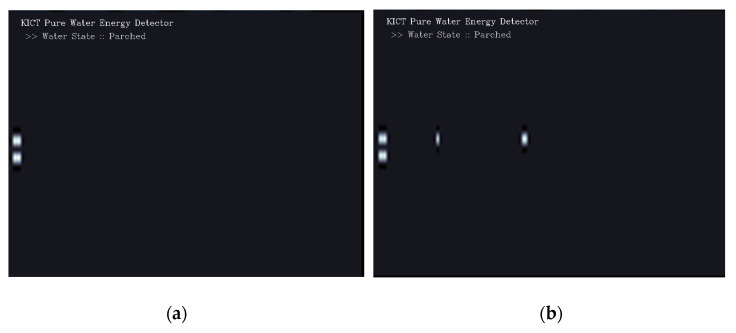

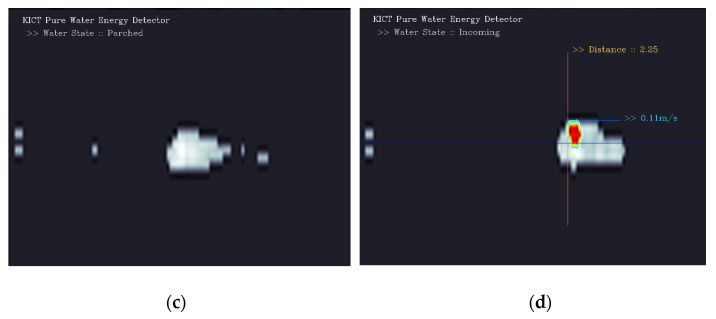
Experimental results on detection of flooding start point and detection delay time: (**a**) just before the flood; (**b**) 1 s after the flood; (**c**) 2 s after the flood; and (**d**) 3 s after the flood.

Figure 15 shows the operating status from the time flooding begins at a specific distance to the time it takes for WAVE-Surf to detect flooding. From the proposed algorithm, the buffering frame N for object filtering and pure flow energy detection was set to N = 5, and WAVE-Surf was set to operate at 1 fps. Figure 15a–d show the pure flow energy detection results 0 s, 1 s, 3 s, and 4 s after the start of flooding, respectively. When the flooding simulation device is not operated, that is, when no flow occurs, the results of WAVE-Surf always show the results shown in Figure 15a. When the flooding simulation device is not operated, that is, when no flow occurs, the results of WAVE-Surf always show the results shown in Figure 15a. From the experimental result in Figure 15d, it is confirmed that flooding was detected exactly 5 s after the flow occurred. Additionally, the areas expressed in achromatic colors in Figure 15 mean that obstacles at that distance were recognized. On the contrary, the area where pure flow energy is detected is expressed in chromatic colors, and only then can it be confirmed that the flow speed and distance are calculated and displayed.

**Figure 16:**
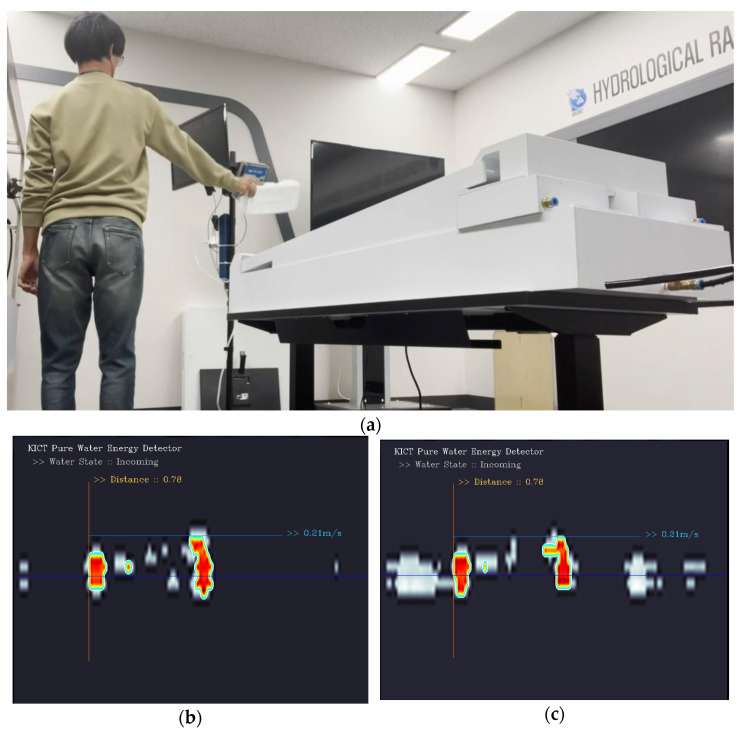
Result of WAVE-Surf flow energy–noise separation through continuous noise generation: (**a**) Continuous noise generation; (**b**) flow energy detection in absence of noise (red area); and (**c**) flow energy detection (red area) with noise signal (gray area).

WAVE-Surf was applied utilizing algorithms that detected only flooding by separating the pure flow energy from the surrounding noise. Several experiments were conducted to separate the flow energy and noise. The results of generating continuous noise between the WAVE-Surf and the flooding simulation device are presented in Figure 16.

The process of monitoring the changes in flow energy observed when noise was continuously generated for 1 min in front of WAVE-Surf is presented in Figure 16a, whereas the result of monitoring the flow energy without noise is depicted in Figure 16b. The red part indicates the observed flow energy when the flooding distance is 0.61 m. The result of generating continuous noise between WAVE-Surf and the water tank at the same distance as in (b) is presented in Figure 16c. The method was programmed to express noise in grey to distinguish between noise signals. The experimental results revealed that the flooding distance was 0.78 m, which was identical to the case without noise, even when continuous noise was generated.

### 4.2. Experimental Results in Outdoor Environments

**Figure 17:**
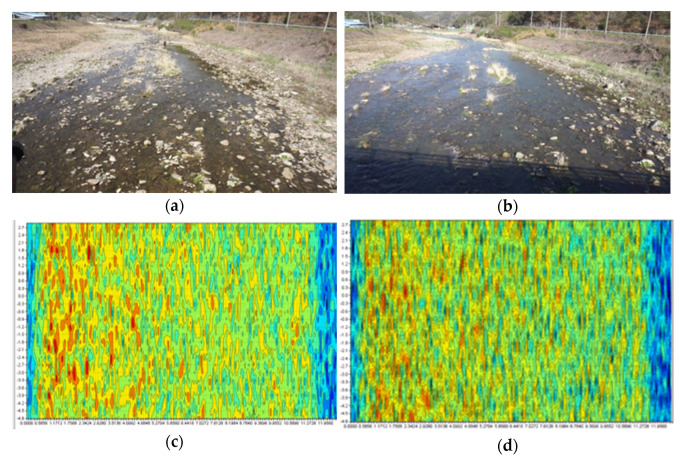

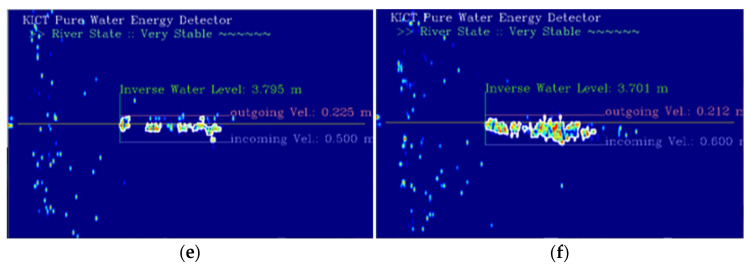
Field tests for performance validation of proposed WAVE-Surf: (**a**,**b**) test environments before rain and two days after rain, respectively; (**c**,**d**) raw range–Doppler maps observed from (**a**,**b**); (**e**,**f**) distances and velocities determined after flow energy detection.

To validate the accuracy and limitation of the proposed WAVE-Surf’s flood detection performance, an experiment was conducted in a real stream before and after precipitation, and the following results were obtained:

Before applying WAVE-Surf to a complex urban environment, the proposed method was tested in a rough environment to validate its sensitivity to energy flow. The environment in which the experiment was conducted, at a height of 4 m, where the water level was less than 30 cm, is presented in Figure 17a,b; Figure 17a illustrates the stream before the rain, and (b) illustrates the stream two weeks after the rain. The stream flow increased owing to rainfall. Graphs of the range–Doppler maps for the original signals observed from the site illustrated in (a) and (b) are presented in Figure 17c,d, respectively. Even after the rain, the streams in (a) and (b) maintained a low water level and slow velocity, where the reflectivity of water energy was lower than or similar to the radar noise; thus, significantly visible values from the range–Doppler maps in (c) and (d) were challenging to determine. Suppose the algorithm proposed in Section 3.2.1 is utilized to detect the flow energy. Accordingly, an accurate water level and velocity can be detected, as illustrated in Figure 17e,f, respectively. The number of frames for the flow energy mask was set to N = 5.

The experimental results in Figure 17e,f indicate that the distances between WAVE-Surf and the water surface are 3.795 and 3.701 m before and after the rain, respectively. Thus, the water level increases by approximately 9 cm because of rain. Further, the maximum velocity increases from 0.5 m/s before the rain to 0.6 m/s after the rain; this experimentally proves the increased velocity from the flow increased by the rain. The field tests presented in Figure 17 reveal that the proposed flood-monitoring technique can detect the water level and velocity with high precision.

Meanwhile, other experiments were conducted in river environments with different water levels and velocities to verify whether the water level and velocity could be accurately detected as river conditions changed.

**Figure 18:**
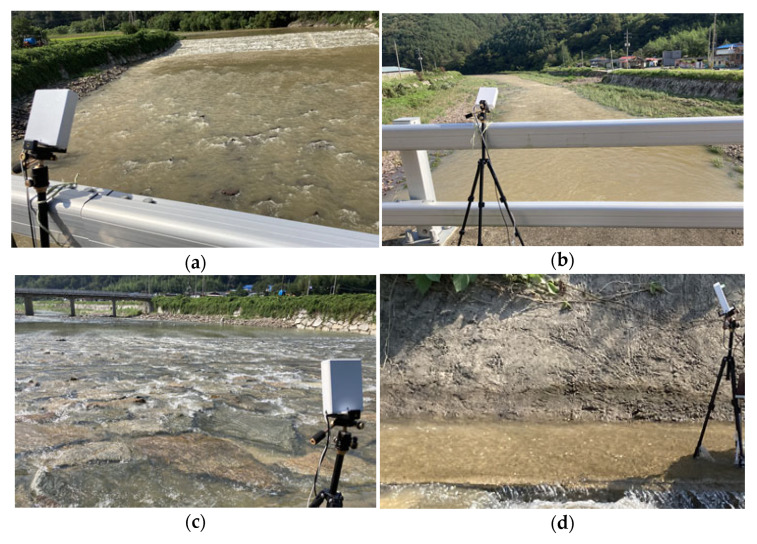
Tests in various river environments to verify the performance of the proposed WAVE-Surf: (**a**,**b**) Upstream and downstream observations from the bridge; (**c**) Observation from a river underwater weir; (**d**) Observations from below the river bank.

In fact, since it was difficult to change the actual river’s water level or velocity rapidly in a short period of time, the observation experiments were performed on a bridge that was relatively far from the river assuming that the river water levels were low, as shown in Figure 18a,b. Conversely, assuming a time when the river’s water level was rising, the experiments were conducted in places where the flow speed was fast and closed to the water surface such as the river’s underwater weir and under the embankment, as shown in Figure 18c,d.

**Figure 19:**
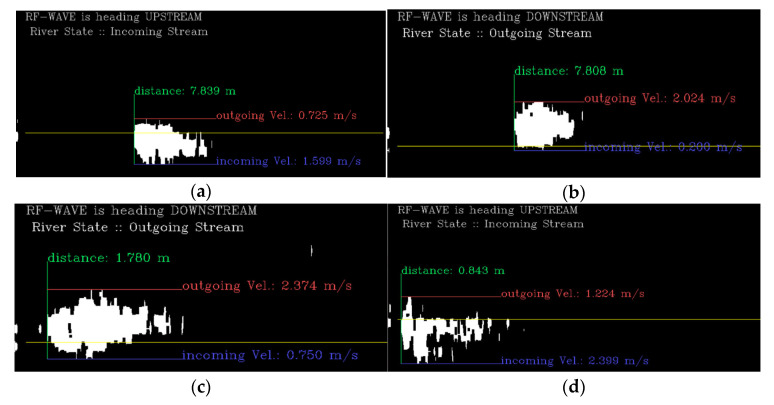
Pure flow energy, stream direction, water level and velocity extracted from each river environment in Figure 17: (**a**) result of Figure 17a, (**b**) result of Figure 17b, (**c**) result of Figure 17c, and (**d**) result of Figure 17d.

The results of calculating the water level, flow velocity, and flow direction using our pure flow energy detection algorithm for the observed data at each location in Figure 18 are shown in Figure 19 below.

From the results in Figure 19a, river information (a vertical distance of 7.84 m and a maximum flow speed in the incoming direction of 1.59 m/s) was obtained on a bridge approximately 8m high from the river. Due to the influence of vortices generated from the topography of the river bottom, a flow speed of 0.73 m/s in the outgoing direction was observed, and on average, the maximum flow speed in the (−) direction was observed to be larger than the flow speed in the (+) direction. From this, it can be seen that the river flows in front of WAVE-Surf. These results were all accurately determined in various environments. Figure 19b shows the results observed in the downstream direction from the same bridge as Figure 18a. The maximum flow speed was 2.02 m/s at a vertical distance of 7.81 m, which was almost the same as upstream.

From these two results, the water level observations of WAVE-Surf were verified from the water level observations upstream and downstream of the bridge and the actual height of the bridge. In addition, the downstream area was observed to have a flow speed that was approximately 0.4 m/s faster than upstream, which is believed to be a change in flow speed related to the scour phenomenon around the bridge pillars and their surroundings.

Figure 19c,d show the observation results obtained by installing WAVE-Surf in the downstream direction above the river’s underwater weir and in the upstream direction under the river embankment, respectively. From the results of Figure 19c,d, when tested under the river’s underwater weir and river embankment, the distances from the water surface were observed to be 1.78 m and 0.84 m, respectively. These results are clearly different from those obtained when tested on a bridge, showing that accurate observation results were obtained. Additionally, due to the influence of large drops caused by surrounding structures, the maximum observed flow velocities were 2.37 m/s and 2.40 m/s, respectively. From these results, the flow velocity was observed to be higher than that observed around the bridge, where the river width was wide and the slope was gentle, making it possible to trust the accuracy of the proposed technology.

**Figure 20:**
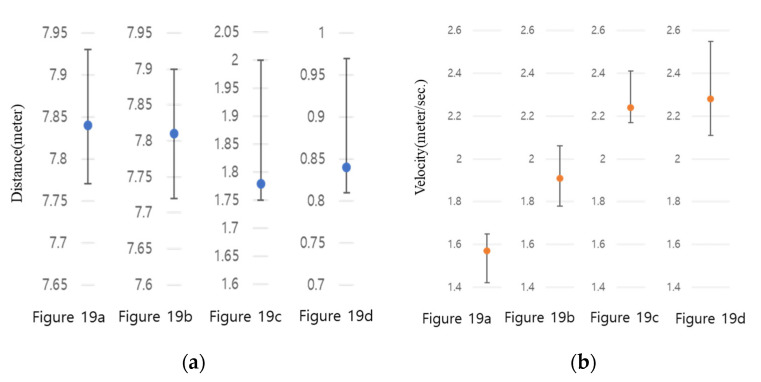
Fluctuation rate of water level and flow rate observed for one hour: (**a**) average, highest, and lowest water level; (**b**) average, highest, and lowest velocity in each experimental environment in Figure 18.

In order to more accurately verify the reliability of observation errors, each of the four cases in Figure 19 was observed for more than 1 h and the maximum, minimum, and average values were calculated, and the deviation of the observed results was analyzed and is summarized in Figure 20.

From the results in Figure 20a, it can be seen that water level changes according to water depth in the environments of Figure 18a,b were stably observed within 10 cm. This water level change error can be viewed as being caused by natural waves occurring on the surface of the river flowing through the observation point. Meanwhile, in the environments shown in Figure 18c,d, the water level fluctuation range was observed to be higher than the average in places where the water depth was shallow and there were many obstacles. These results show that the proposed WAVE-Surf technology performed stably in terms of water level observation. Figure 20b shows the fluctuation range of flow velocities observed in the environments of Figure 18. As with the water level observations, it was confirmed that an overall stable flow rate was observed.

### 4.3. Discussion

Our WAVE-Surf technology was proposed to monitor real-time flooding and provide notifications about risks by applying it to rivers, low-lying areas in urban areas, and underground facilities with complex structures, as global flood damage is increasing due to rapid climate change. From the experimental results, it was verified that WAVE-Surf can stably measure water level and flow speed in various environments containing obstacles. In this chapter, we will discuss the applicability of the proposed technology through a simple example.

The proposed WAVE-Surf technology is designed as a small, low-power embedded system. Additionally, flooding information can be provided with a very small amount of data. (If you know the installation location of WAVE-Surf, only the terminal ID, water level, and flow rate can be transmitted when a change in water level or flow rate occurs.)

**Figure 21:**
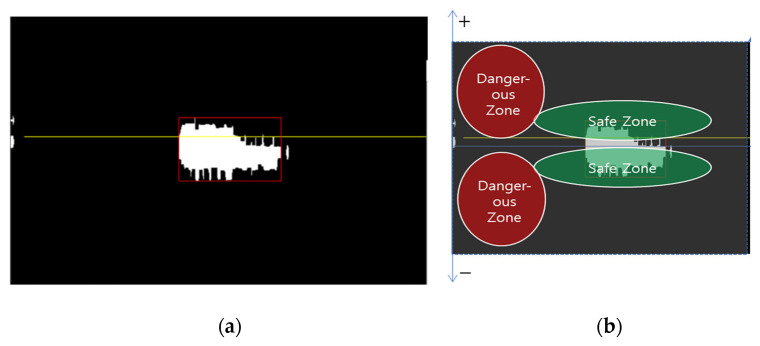
Concept of flood risk determination using WAVE-Surf: (**a**) example of detected pure flow energy; (**b**) flood risk on the range–Doppler map.

**Figure 22:**
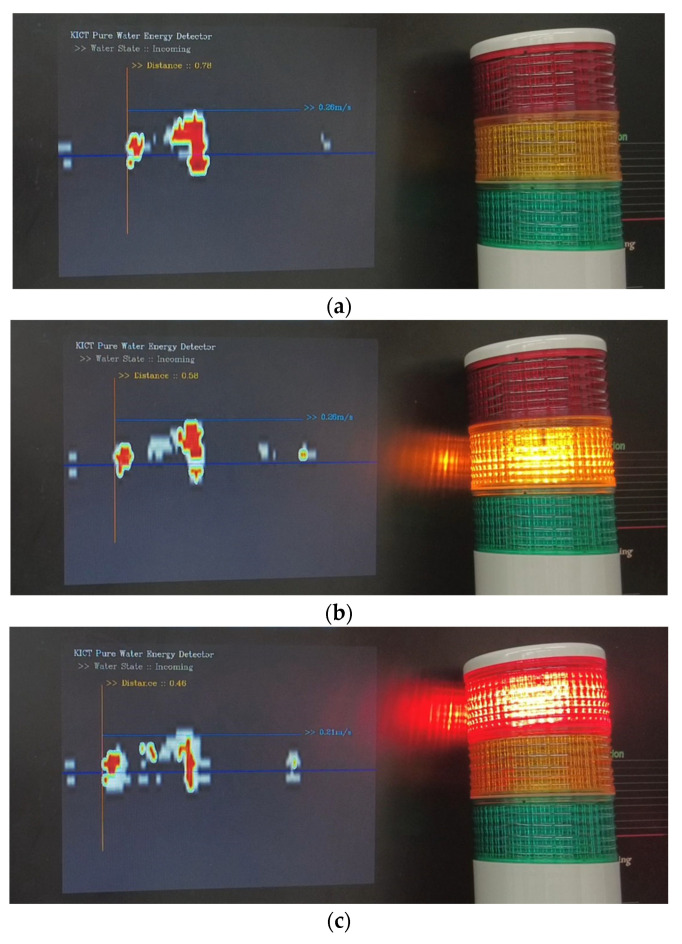
Simulation of WAVE-Surf observation data and alarm interworking for risk decision in Figure 21b: (**a**) normal draining condition (observation distance 78 cm); (**b**) flood beginning (observation distance 58 cm); (**c**) flood in progress (observation distance 46 cm).

From Figure 20, it is confirmed that the proposed WAVE-Surf technology stably detects water level and flow speed without significant error. Accordingly, we would like to present a method to simply determine the risk of flooding by determining the risk limits for flow speed and distance in advance according to the environment in which WAVE-Surf is installed. Figure 21a below shows an example where pure flow energy is shown according to distance and flow speed on the range–Doppler map, and Figure 22b shows an example where WAVE-Surf automatically determines the risk depending on this energy’s location on the map.

An example of warning of danger using the method shown in Figure 21 is shown in Figure 22. A rise in water level was simulated by varying the height of the device of Figure 12, and three risk levels were set in advance to issue an alarm.

Following the risk decision, we would like to discuss the possibility of real-time monitoring and a digital twin of WAVE-Surf. We have established a simple digital twin environment in our laboratory and are constantly operating WAVE-Surf in the laboratory environment. WAVE-Surf transmits the observed water level and velocity data to the IoT server every second to be stored in a database, whereas the control center constantly monitors the transmitted data and performs digital pairing of the water level and velocity of the virtual river system. The changes in the virtual water level and velocity data were implemented employing the Shader of OpenGL and a digital elevation model (DEM) of the virtual city for real-time synchronization in the virtual river.

**Figure 23:**
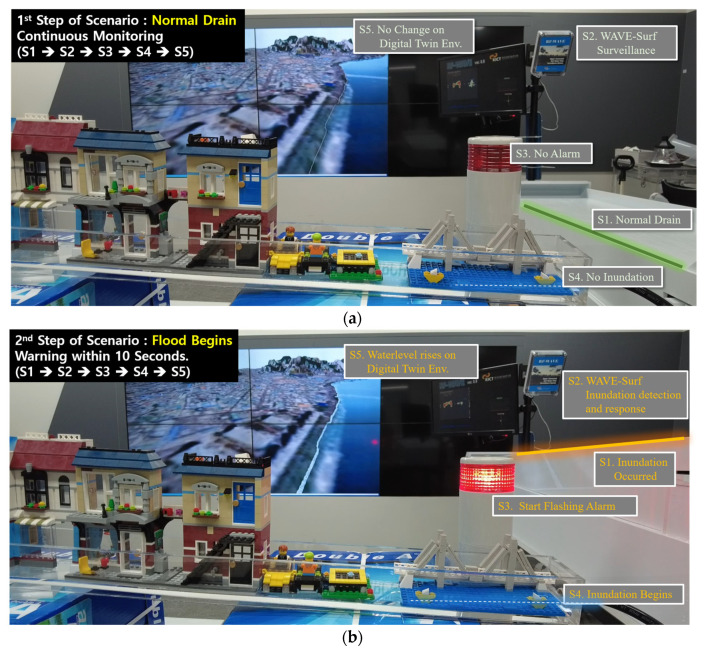

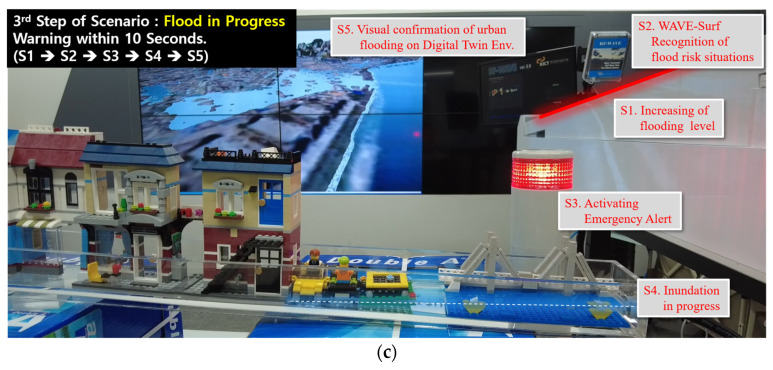
Urban flood-monitoring and response scenarios for real-time WAVE-Surf observations: (**a**) normal draining condition; (**b**) flood beginning; (**c**) flood in progress.

The results of simulating the steps of each scenario of the urban flood-monitoring system according to the water level and velocity observed by WAVE-Surf in real time are presented in Figure 23.

Through the digital twin results of WAVE-Surf, it is expected that the actual control center will be able to provide various services to the public, such as continuous emergency response and flood analysis/management.

## 5. Conclusions

This study proposed a real-time urban flood-monitoring technology as an urban disaster prevention measure for sustainable smart cities. The proposed WAVE-Surf was designed based on IoT technology and can be easily operated via installation in complicated urban environments encompassing lowlands, underpasses, underground facilities, and hydrophilic parks in cities. Unlike conventional macroscopic flooding models designed based on the precipitation amount and geographic information systems, the proposed technology was experimentally validated to be capable of detecting flooding with a high precision of 1 cm by measuring the water level and velocity through the detection of pure flow energy from the Doppler velocity and reflectivity of a radar sensor utilizing signal-processing algorithms. Unlike previous flooding sensors, WAVE-Surf does not require extensive infrastructure construction for installation, and a non-contact-type subminiature sensor platform can be easily installed with fewer spatial limitations. In particular, WAVE-Surf uses radar signals, so unlike CCTV surveillance, it is not affected by lighting and can guarantee a certain level of reliability from obstacles blocking its view. Because flooding can be monitored within the radar observation range, its installation flexibility is higher than that of contact-type flood sensors, and because it is installed in a mounted or hanging manner, there are fewer restrictions on operation in indoor and outdoor environments.

The simulation revealed that regions and roads prone to urban flooding can be monitored constantly, direct warnings can be given as emergency response measures to assist people in evacuating or operating shield skirts, and actions can be taken preemptively through public and private facilities, as relevant data are transmitted to the control center in real time. As this technology can monitor the flood level in units of 1 cm, its usability as an original technology for predicting and preventing flooding was validated using a high-resolution flooding digital twin.

In addition, the proposed technology corresponds with the promotional strategy of “People-Smart Sustainable Cities” proposed by UNECE [13], where people can enjoy the benefits of a more livable and safer city. Suppose that the proposed WAVE-Surf platform can be further miniaturized by optimizing hardware and software while reducing the supply cost. Accordingly, a smart road flood-management system can be actualized by linking and converging it with intelligent CCTV operation for various purposes in smart cities. Finally, by building a smart city digital twin river network based on WAVE-Surf, it is expected to contribute to securing water disaster response technology for sustainable smart city life. Additionally, we plan to test this technology by applying it to an actual urban river site next year. Through this demonstration, we plan to periodically receive real-time weather information provided by open weather APIs, etc., using the IoT function installed in WAVE-Surf. We will conduct research on flood prediction and the identification of floods’ causes through continuous data acquisition, technology improvement, and AI learning.

## Figures and Tables

**Table 1:** Various types of water meters according to operation principle.

Division	Type	Description
Direct reading	Staff gauge	Water level readings indicated by a staff gauge
Float	Float	Water level read by the flute in line with buoyancy
	Reed switch	Reed contacts utilized by the buzzer
Pressure	Differential pressure	Atmospheric pressure utilizing differential pressure measurement
	Air purge	Air pressure utilization
Electronic	Electrostatic capacity	Capacitance measurement utilizing the dielectric constant of the aforementioned liquid
	Electrode	Electrical conductivity measured by utilizing the electrodes
Ultrasonic	Ultrasonic wave	Ultrasonic transit time measurement
	Sound wave	Transmission time measurement of the sound wave
Radiation	Irradiation	Utilization of radiation-reflected waves
	Transmission	Radiation transmittance measurement
Microwave		Transmission propagation time measurement of the microwave
Laser		Transmission time difference measurement of the laser
Radar		Echo time difference measurements of radar signals

## Data Availability

Data are contained within the article.
